# Special Meeting of the New York State Medical Association

**Published:** 1904-05

**Authors:** 


### SOCIETY PROCEEDINGS.

### Special Meeting of the New York State Medical Association.

*Held at the New York Academy of Medicine, March 21, 1904.*

From *New York State Journal of Medicine*, April, 1904.

THE meeting was called to order at 8.35 p. m., the president, Dr. William H. Thornton, of Buffalo, in the chair. The secretary, Dr. Guy Davenport Lombard, stated that the purpose for which we meet this evening is in answer to a request addressed to the president, and is as follows:

NEW YORK, Feb. 16, 1904.

DR. WILLIAM H. THORNTON, President, The New York State Medical Association, 572 Niagara Street, Buffalo, N. Y.

*Dear Doctor*—We, the undersigned fellows of The New York State Medical Association, request that you call a special meeting of all the members of the association, to be held at the Academy of Medicine, 17 West 43d street, New York City, on March 21, 1904, at 8 p. m. The object of the meeting is to take action upon the following:

"*Resolved,* That the report of the joint committee of conference be accepted, and that the proposed agreement for the consolidation of the Medical Society of the State of New York and The New York State Medical Association be, and the same is, hereby approved, and the president of the association is hereby authorised and directed to execute the same in the name and behalf of the association, and the secretary is hereby authorised and directed to affix the corporate seal thereto; and be it further

"*Resolved,* That the committee of the association heretofore appointed for the purpose of bringing about the consolidation, namely, Dr. E. Eliot Harris, Dr. Julius C. Bierwirth, Dr. Alexander Lambert, Dr. Parker Syms and Dr. Wisner R. Townsend, be, and they are, hereby continued as such committee, with full power and authority to do whatever may be necessary to carry the agreement into effect."

And also to ask the county medical associations in affiliation with The New York State Medical Association to ratify the agreement.

Very truly, (Signed),

E. ELIOT HARRIS,

PARKER SYMS,

J. C. BIERWIRTH,

ALEXANDER LAMBERT,

WISNER R. TOWNSEND,

JOS. D. BRYANT,

CHARLES G. STOCKTON,

C. C. FREDERICK,

ALLEN A. JONES,

And 29 others.

Dr. Bryant rose to offer a resolution, but at the request of the president it was held over until after the reading of the report of the joint committee of conference by Dr. E. Eliot Harris, chairman of the committee on conference of The New York State Medical Association. Dr. Harris stated that as his report was very lengthy the reading might be suspended at any time if the members so requested. The report of the committee is as follows:

*To the Council Fellows and Members of The New York State Medical Association:*

Your committee appointed and empowered to confer and unite with a committee of the Medical Society of the State of New York, for the purpose of forming a union of the two state medical organisations, respectfully submits the following report:

The first communication the committee received was from your secretary.

Dr. E. Eliot Harris, Chairman, Committee on Conference, 33 West 93d street, New York.

*Dear Doctor Harris*—I have the honor to inform you that at a meeting of the counsel and fellows of The New York State Medical Association, held at the Academy of Medicine, on October 1, 1903, you were appointed chairman of the committee on conference, by the president, with power to do whatever is necessary and expedient to bring about a union of The New York State Medical Association and the Medical Society of the State of New York in a just and equitable way.

I am enclosing herewith the text and preamble and resolution, following which your appointment took place.

Yours very sincerely,
Guy Davenport Lombard,
Secretary.

The following are the resolutions:

Whereas, The members of The New York State Medical Association desire a union of the medical profession of the state of New York, and

Whereas, It is deemed expedient for the attainment of this purpose, to make further effort to bring together The New York State Medical Association and the Medical Society of the State of New York.

*Resolved,* That a committee of five be appointed by the chair, and said committee is hereby empowered to do whatever is necessary and expedient to bring about such a union in a just and equitable manner.

*Resolved,* That the committee so empowered may confer, coöperate and unite with a committee of the Medical Society of the State of New York, for the purpose of forming said union of the two state medical organisations.

*Resolved,* That a copy of these resolutions be transmitted to the secretary of the Medical Society of the State of New York, with a request that their conference committee be granted similar power.

The committee appointed in accordance with this resolution: E. Eliot Harris, chairman; Julius C. Bierwirth, Alexander Lambert, Parker Syms, Wisner R. Townsend.

OFFICE OF THE SECRETARY OF THE MEDICAL SOCIETY OF THE STATE OF NEW YORK.

ALBANY, N. Y., October 14, 1903.

GUY DAVENPORT LOMBARD, Secretary, The New York State Medical Association.

*Dear Sir*—At a duly called meeting of the Medical Society of the State of New York, held in the city of New York, on October 13, 1903, the following was adopted unanimously:

WHEREAS, The New York State Medical Association, at a recent special meeting, duly assembled, has by unanimous vote appointed a committee with full power to meet a similar committee of the Medical Society of the State of New York, to arrange for the unification of the two organisations under the corporate name of the Medical Society of the State of New York, therefore be it

*Resolved,* That the committee on conference of the Medical Society of the State of New York, already appointed, be given powers equal to and commensurate with those granted the committee created by The New York State Medical Association, for the purpose of unifying the two state medical bodies in the Medical Society of the State of New York.

I have the honor to transmit this to you, as the action of this society, pursuant to that of the association, of which you are secretary, referred to in the preamble of the resolution.

Respectfully,

FREDERIC C. CURTIS,
Secretary.

The chairmen of the committees on conference arranged for the first joint meeting, which was held at the Academy of Medicine, on the afternoon and evening of October 30, 1903. At this meeting the joint committee on conference was organised by electing Dr. A. Jacobi chairman and Dr. Wisner R. Townsend secretary. A draft of a bill to be presented to the legislature was read and adopted. A sub-committee of three, consisting of Dr. A. Jacobi, Dr. E. Eliot Harris and Dr. George R. Fowler, was empowered to employ legal counsel to arrange the draft of the proposed bill, for the consolidation of the two state medical bodies, in proper legal form, to be introduced in the legislature of 1904. The sub-committee unanimously agreed to select as counsel, Mr. Howard Van Sinderen, of New York, a lawyer well known in the medical profession, he being the counsel of the New York Academy of Medicine.

Mr. Van Sinderen framed in legal form a draft of the proposed bill, "Entitled An Act" for the consolidation of The New York State Medical Association and the Medical Society of the State of New York, and accompanied the proposed bill with a written opinion, attacking its constitutionality, on the ground that mandatory legislation affecting the vested rights of the members of the two state medical bodies was illegal.

Several members of the joint committee on conference secured opinions from constitutional lawyers in this state, and they all confirmed Mr. Van Sinderen's opinion as to the unconstitutionality of the proposed mandatory legislation. Since mandatory legislation was declared illegal, Mr. Van Sinderen suggested a plan of securing permissive legislation, and the sub-committee, with his aid, laid before the joint conference committee a bill to be introduced into the legislature, entitled, "An Act, to authorise the consolidation of the Medical Society of the State of New York and The New York State Medical Association," which was adopted unanimously by the joint conference committee, at a meeting held at the New York Academy of Medicine on January 5, 1904. This so-called permissive act was passed by both houses of the legislature, and was signed by the Governor on January 21, 1904. (Then followed the unanimous report of the committee, which is like that presented to the Medical Society of the State of New York, January 26, 1904.)

Respectfully submitted,

E. ELIOT HARRIS, Chairman;
JULIUS C. BIERWIRTH,
ALEXANDER LAMBERT,
PARKER SYMS,
WISNER R. TOWNSEND.

After the report of the joint committee of conference had been partially read, Dr. Bryant moved that the reading be discontinued, on the ground that the report had already been in the hands of every member of the association, and the members were therefore familiar with it. Carried. Dr. Ferguson then asked if in the report there were reasons given for the action advised, to which Dr. Harris answered that it was the official report of the "act" which had passed the legislature, and was the constitution and by-laws of The New York State Medical Association. Dr. Bryant then offered the following resolution:

"*Resolved,* That the report of the joint committee of conference be accepted, and that the proposed agreement for the consolidation of the Medical Society of the State of New York and The New York State Medical Association be and the same is hereby approved, and the president of the association is hereby authorised and directed to execute the same in the name and behalf of the association, and the secretary is hereby authorised and directed to affix the corporate seal thereto; and be it further

"*Resolved,* That the committee of the association heretofore appointed for the purpose of bringing about the consolidation—namely, Drs. E. Eliot Harris, Julius C. Bierwirth, Alexander Lambert, Parker Syms and Wisner R. Townsend, be and they are hereby continued as such committee, with full power and authority to do whatever may be necessary to carry the agreement into effect."

The president, Dr. Thornton, then stated that in considering these questions it should be borne in mind that the committee appointed by President Wiggin, was one which, perhaps as well as any committee could have done, had commanded the respect and confidence of the entire association. The report of the joint committee of conference, as it had been partially read by the chairman of the committee on conference of the state association, represents in a very small degree the tremendous amount of work involved in the consideration of these questions, which has required a great sacrifice of time and patience, courtesy and energy on the part of the members of the joint committee. A matter of such great importance as the question of reconciling so many diverse interests, cannot be possibly accomplished without concessions and sacrifices by both parties, and I feel that the members of the committee on conference will have represented the interests of The New York State Medical Association, in that they have been able to carry out as far as possible the wishes of the association. In considering these questions I also feel that we should consider not only what is for our present interests, not our feelings for the present time, but should take into consideration the question of the future welfare of the medical profession of the state of New York, and look upon this action, not as it appears for the present, but as it will appear in future years. The question of adoption of the resolutions is now open for discussion by the members of the association.

Dr. E. D. Ferguson, of Troy, said that he would like to have any one favoring the report say whether the plan of union is for the best interests of the association, and if it carried out the statement of the association in the resolution, that the plan of union should be a "just and equitable" one for the association would be true. The question was not answered. I have a word to say if no one wishes to speak, and I trust that the time will be given to me to say it by the association.

It is twenty-one years that I have worked for The New York State Medical Association, and I feel that I have the right to ask for a few minutes—ten, fifteen, possibly twenty. I am not here tonight, Mr. Chairman, to vote against the action of this committee, for I know what an awkward position would be created by the rejection of their report, but I am here to express my sentiments, and not mine only, but those of a large majority of the association, that we have not been justly treated, and in giving up what this association has stood for in this movement toward a union, we know that we have been snared. The American Medical Association years ago let the old members of the state society come and read their papers, if they were so inclined, but when the line was drawn at the meeting of the American Medical Association at Denver, and it was said that no one could be in proper affiliation from their state without being a member of the association representing the American Medical Association in that state, from that moment the membership of the association in the state of New York increased, and from that moment a desire to get into the American Medical Association, in some form, shape or fashion, was constantly being pressed. There was one door that was open, one door that was honorable, and that door was through membership in The New York State Medical Association. Many men came and took their membership and took it honestly. Then there began to be a struggle, I can see it now, but the state association stood squarely upon its basis, and the Medical Society of the State of New York came and asked for union. They did this over two years ago. We did not ask for it, but we were willing to give it. And after the meeting at Saratoga, the committee of the Medical Society of the State of New York expressed their willingness to accept the conditions as they were, but they did not carry it out. When our committee was first appointed I was especially urged not to have upon the committee any of the old men, that there should be new blood in the committee. There were no old warriors on our committee, but the committee of the Medical Society of the State of New York was made of ex-presidents and old members, and I did not like it when I heard it. At the meeting at New Orleans there was a movement made for a new code of ethics; God knows we did not need it; it was strong enough as it was for any honest man, and no one can make one strong enough for a dishonest one, and now the code is not part of the union.

What are the terms of union? They are the plan of organisation as planned by the American Medical Association. It is not the original plan of organisation of The New York State Medical Association; it may not be the plan that is best for any one; it is not proved yet that the bodies will not be controlled by cliques. We have simply yielded everything, and I would ask the committee the question if their plan is just and equitable to the state association? And I wish to say that from the very bottom of my heart I feel the injustice of it. I feel that The New York State Medical Association has simply been bereft of everything that it stood for. The committee had the power to do this, it is true, but if the committee had asked for different terms of union those terms would have been granted, with more honor to us, and they know it.

And now to the committee itself. For this betrayal of the association, I am impelled to utter the words that, to the best of my memory, Shakespeare put in the mouth of the outraged queen:

> "Thou little valiant, great in villainy!
> Thou ever strong upon the stronger side!
> Thou Fortune's champion, that dost never fight
> But when her humorous ladyship is by
> To teach thee safety! thou art perjured, too,
> And sooth'st up greatness; What a fool art thou
> A ramping fool; to brag and stamp and swear
> Upon my party! Thou cold-blooded slave,
> Hast thou not spoke like thunder on my side?
> Been sworn my soldier? Bidding me depend upon
> Thy stars, thy fortune and thy strength?
> And dost thou now fall over to my foes?
> Thou wear a lion's hide! doff it for shame,
> And hang a calf's skin on those recreant limbs."

I am done. My association work is finished. I cannot say good-bye to you, the word means too much, I cannot say farewell for the association is about to die, and I am even debarred the thin Gallicism of au revoir. Nothing is left to say, I go.

Dr. Bryant then moved that the county associations in affiliation with the state association be requested to ratify the action of the state association. Both resolutions were unanimously carried.

Dr. Thornton then acknowledged with thanks the courtesy of the New York County Medical Association for its kindness, and moved that if there was no further business before the association that the meeting be adjourned.

